# Sources of Human Immunodeficiency Virus Infections Among Men Who Have Sex With Men With a Migration Background: A Viral Phylogenetic Case Study in Amsterdam, The Netherlands

**DOI:** 10.1093/infdis/jiae267

**Published:** 2024-07-08

**Authors:** Alexandra Blenkinsop, Nikos Pantazis, Evangelia Georgia Kostaki, Lysandros Sofocleous, Ard van Sighem, Daniela Bezemer, Thijs van de Laar, Marc van der Valk, Peter Reiss, Godelieve de Bree, Oliver Ratmann

**Affiliations:** Department of Mathematics, Imperial College London, United Kingdom; Department of Hygiene, Epidemiology and Medical Statistics, Medical School, National and Kapodistrian University of Athens, Greece; Department of Hygiene, Epidemiology and Medical Statistics, Medical School, National and Kapodistrian University of Athens, Greece; Department of Mathematics, Imperial College London, United Kingdom; Stichting HIV Monitoring, The Netherlands; Stichting HIV Monitoring, The Netherlands; Department of Donor Medicine Research, Sanquin, The Netherlands; Stichting HIV Monitoring, The Netherlands; Amsterdam Institute for Infection and Immunity, Amsterdam University Medical Center, The Netherlands; Amsterdam Institute for Global Health and Development, The Netherlands; Department of Global Health, Amsterdam University Medical Center, University of Amsterdam, The Netherlands; Amsterdam Institute for Infection and Immunity, Amsterdam University Medical Center, The Netherlands; Amsterdam Institute for Global Health and Development, The Netherlands; Department of Mathematics, Imperial College London, United Kingdom

**Keywords:** HIV, phylogenetics, migrants, molecular source attribution, shifting transmission dynamics

## Abstract

**Background:**

Men and women with a migration background comprise an increasing proportion of incident human immunodeficiency virus (HIV) cases across Western Europe.

**Methods:**

To characterize sources of transmission in local transmission chains, we used partial HIV consensus sequences with linked demographic and clinical data from the opt-out AIDS Therapy Evaluation in the Netherlands (ATHENA) cohort of people with HIV in the Netherlands and identified phylogenetically and epidemiologically possible HIV transmission pairs in Amsterdam. We interpreted these in the context of estimated infection dates, and quantified population-level sources of transmission to foreign-born and Dutch-born Amsterdam men who have sex with men (MSM) within Amsterdam transmission chains.

**Results:**

We estimate that Dutch-born MSM were the predominant sources of infections among all Amsterdam MSM who acquired their infection locally in 2010–2021, and among almost all foreign-born Amsterdam MSM subpopulations. Stratifying by 2-year intervals indicated time trends in transmission dynamics, with a majority of infections originating from foreign-born MSM since 2016, although uncertainty ranges remained wide.

**Conclusions:**

Native-born MSM have predominantly driven HIV transmissions in Amsterdam in 2010–2021. However, in the context of rapidly declining incidence in Amsterdam, the contribution from foreign-born MSM living in Amsterdam is increasing, with some evidence that most local transmissions have been from foreign-born Amsterdam MSM since 2016.

## Teaser

Migrants living in Western Europe comprise an increasing proportion of incident cases of HIV. We use viral sequence data contextualized by time-since-infection estimates to characterize sources of HIV transmissions in 2010–2021 among men who have sex with men living in Amsterdam by place of birth.

## Graphical Abstract

**Graphical Abstract:**
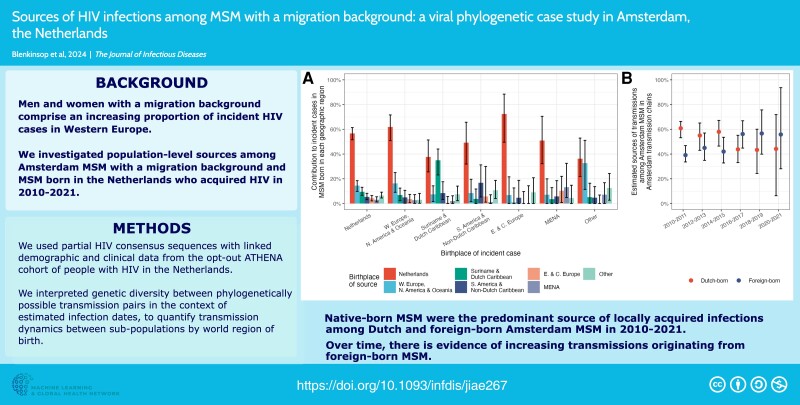

While overall new cases of human immunodeficiency virus (HIV) among men who have sex with men (MSM) have been declining across Europe over the past 10 years, new diagnoses among foreign-born MSM have been increasing [1], suggesting gaps in HIV care and prevention services. Several recent studies characterized the frequency of postmigration HIV acquisition among MSM with a migration background (MSM-MB) across Europe together with risk factors, finding that the majority of recruited MSM-MB acquired infection postmigration in 2013–2015 [2] and 2021–2022 [3]. These findings were based on MSM-MB recruited cross-sectionally in clinic settings, and estimates into the timing of infection relative to migration were derived from biomarker and questionnaire data. HIV pathogen surveillance data from the Netherlands corroborated these trends, with a focus on Dutch-born and foreign-born individuals with HIV [4]. Here, we estimate who the source populations of local HIV acquisitions have been in 2010–2021, and focus on the sources among Amsterdam MSM stratified by world region of birth, who acquired HIV locally within Amsterdam transmission chains.

Amsterdam, the capital of the Netherlands, was one of the early epicenters of the HIV epidemic in Europe and has to date remained an HIV hotspot in the Netherlands [4]. In 2014, the HIV Transmission Elimination Amsterdam (H-TEAM) Consortium was established to develop an evidence-led response to eliminate HIV within the city (www.hteam.nl). Following the implementation of several successful interventions [5, 6], Amsterdam has surpassed Joint United Nations Programme on HIV/AIDS (UNAIDS) targets, with 97% of individuals knowing their status, 95% of diagnosed individuals on treatment, and 96% of treated individuals virally suppressed [7]. Yet, despite first-generation migrants comprising only a third of the general population in Amsterdam, 56% of new diagnoses among MSM in 2014–2019 were in men born outside of the Netherlands [4].

Several previous European studies identified growing proportions of non-B subtypes among MSM [8], suggesting that transmission dynamics may be increasingly originating from non-native source populations, since non-B subtypes are more prevalent than subtype B outside Europe. It is unclear if these trends reflect a constant rate of external importations amid declining local transmission, an increasing rate of importations, or increased local transmission from MSM with non-B subtypes. Phylodynamic analyses can help resolve these alternative hypotheses, especially when comprehensive background sequences are used to disentangle local, growing transmission chains from importations [4, 9], with data from the United Kingdom (UK) up to 2015 [9] suggesting that the majority of non-White MSM with HIV acquired infection from a White transmission source. However estimates were based on subtype B sequences only, so it remains unclear if these generalize to all MSM.

Here, we applied pathogen phylodynamic analyses across predominant subtypes to estimate the sources of local HIV infection among Amsterdam MSM in 2010–2021. We explicitly focused on the sources of the 67% of HIV infections in local transmission chains among individuals who are or have been Amsterdam residents, as these transmissions could potentially have been averted through local prevention strategies [4]. With similar trends in incidence observed in migrant MSM across France, the UK, and other European countries [1, 10, 11], our study may provide useful insights beyond Amsterdam.

## METHODS

### The ATHENA Cohort Study

Data were collected as part of the open, opt-out AIDS Therapy Evaluation in the Netherlands (ATHENA) observational cohort study of people with HIV, with earliest date of diagnosis on 12 June 1981 [12]. People entering HIV care receive written material about ATHENA, after which they consent participation or opt out. As of May 2022, 2.6% of eligible participants opted out and 6.9% were lost to follow-up [7]. For this study, data were frozen on 1 February 2022 and comprised pathogen genomic, epidemiological, and clinical data for MSM ever resident in Amsterdam based on postal codes (PC4) of participants’ address at registration or registration updates. MSM were grouped into geographic regions of birth corresponding to the primary migrant groups among MSM living with HIV in Amsterdam: Western Europe; North America and Oceania; Eastern and Central Europe; Suriname and the Dutch Caribbean; South America and the non-Dutch Caribbean; the Middle East and North Africa; or other world regions (Supplementary Figure 1).

### Infection Dates and HIV Incidence

Our study population comprised Amsterdam MSM with an estimated infection date during 1 January 2010 to 31 December 2021. Specifically, we used a Bayesian model fitted to declines in CD4 counts and viral load trajectories from 19 788 seroconverters of the Concerted Action on SeroConversion to AIDS and Death in Europe (CASCADE) collaboration [13], and then predicted infection dates from the model using longitudinal biomarker study data [4]. Incidence among Amsterdam MSM was estimated after accounting for estimated probabilities of remaining undiagnosed by database freeze using a Bayesian time-to-diagnosis model [4]. Annual HIV prevalence was estimated by summing incidence and subtracting individuals known to have died. See the Supplementary Material for details on this approach, as well as on all other methods described below.

### Subtype Frequencies

Partial HIV-1 polymerase (pol) sequences spanning the protease and reverse-transcriptase encoding regions were obtained for 40% of participants in the Netherlands, and 55% of participants with an Amsterdam postcode as part of routine drug-resistance screening, with mean fragment lengths of 1248 base pairs (standard deviation, 166). We estimated subtype frequencies as an indicator of transmission sources, using as denominator the estimated number of incident Amsterdam MSM per year (accounting for undiagnosed infections by database freeze), and as numerator the number of sequenced incident Amsterdam MSM with B or non-B subtypes (accounting for undiagnosed/unsequenced incident cases). The latter was estimated in 2 ways: assuming subtype distributions were as seen among diagnosed and sequenced cases, or assuming conservatively for our hypotheses that all undiagnosed incident cases had subtype B.

### Phylogeographic Analysis

More than 80 000 international *pol* sequences were downloaded from the Los Alamos database (www.hiv.lanl.gov) and the closest sequences to ATHENA sequences were selected with BLAST (v2.10.0) [14] for phylogenetic background. ATHENA sequences were aligned with Virulign (v1.0.1) [15] and MAFFT (v7.453) [16] following manual curation, and subtyped using COMET (v2.3) [17] and REGA (v3.0) [18]. Phylogenetic trees were inferred separately with FastTree (v2.1.11) [19] for all major subtypes and circulating recombinant forms (CRFs) in Amsterdam (B, 01AE, 02AG, A1, C, F1, G, D, 06cpx) under a general time-reversible nucleotide substitution model to allow for heterogeneous substitution rates across sites, excluding other subtypes/CRFs due to small sequence numbers. Phylogenies were subsequently re-rooted against a background reference sequence of a closely related subtype or CRF. Groups of lineages sharing a common ancestral state (other Amsterdam risk groups, other ATHENA participants from the rest of the Netherlands, other individuals stratified by world region of birth, undetermined state) were identified with phyloscanner (v1.8.0) [20] using a modified maximum parsimony algorithm that mitigates unequal sampling densities of hosts [21].

### Possible Transmission Pairs

For each incident Amsterdam MSM since 2010 with an HIV sequence, we considered any Amsterdam MSM ATHENA participant with estimated infection date prior to that of the incident case as a potential source. Following ancestral state reconstruction, we retained those potential sources in the same phylogeographically reconstructed Amsterdam transmission chain as the incident case as phylogenetically possible sources [22]. We excluded phylogenetically possible pairs if metadata indicated the source had died or not yet migrated to the Netherlands prior to the estimated infection date of the recipient; if the potential transmitter was likely noninfectious based on a viral load <200 copies/mL [23] on the estimated infection date of the recipient as derived from LOESS (locally estimated scatterplot smoothing) fits through longitudinal viral load measurements [22]; and if the estimated time elapsed was >16 years, because under typical disease progression it is unlikely for 2 individuals to remain undiagnosed (thus unsampled) for longer than 8 years [24]. Unlike most clustering analyses, we thus retained data from all phylogeographically identified Amsterdam MSM transmission chains of size 2 or larger, and avoided branch length cut-offs, which are confounded by time since infection [25].

### Source Attribution

We estimated population-level transmission flows and sources of transmissions from the pairs using a Bayesian mixture model that harnesses both phylogenetic data and time elapsed from the estimated infection date and the sequence sampling date of both the recipient and source [26]. The model classifies potential transmission pairs probabilistically to being a transmission pair or not, leveraging information from estimated infection times to ascertain that observed patristic distances are consistent with the number of mutations expected under the HIV evolutionary clock. Potential transmission pairs with large patristic distances are thus not necessarily penalized when the time elapsed between the transmission event and the sampling dates is also large.

As input, the model primarily requires an estimate of the HIV evolutionary clock, which we obtained by fitting a nonlinear meta-analysis clock model to 2807 data points of patristic distances and time elapsed from confirmed transmission pairs in a known subtype C Belgian transmission chain involving 8 individuals [27, 28]. Numerical inference was done with Stan (cmdstanr v2.28.1), with 4 chains of 2500 iterations. From the fitted model, we extracted posterior transmission pair probabilities such that each incident case can have at most 1 likely source and such that the true source may not be present in the data, with no restriction on the number of incident cases a source could have infected. We then estimated population-level transmission flows by aggregating over posterior transmission probabilities for each incident case, adjusting for undiagnosed and unsequenced incident cases in 2010–2021 by multiplying transmission pair probabilities with inverse probability sampling weights.

Uncertainty in infection time estimates and phylogeographic reconstruction was accounted for by bootstrap sampling sequence alignment positions and infection time estimates, and repeating analyses.

## RESULTS

### Increasing Frequency of Non-B Subtypes Is Shown Among Amsterdam MSM

**Figure 1.**
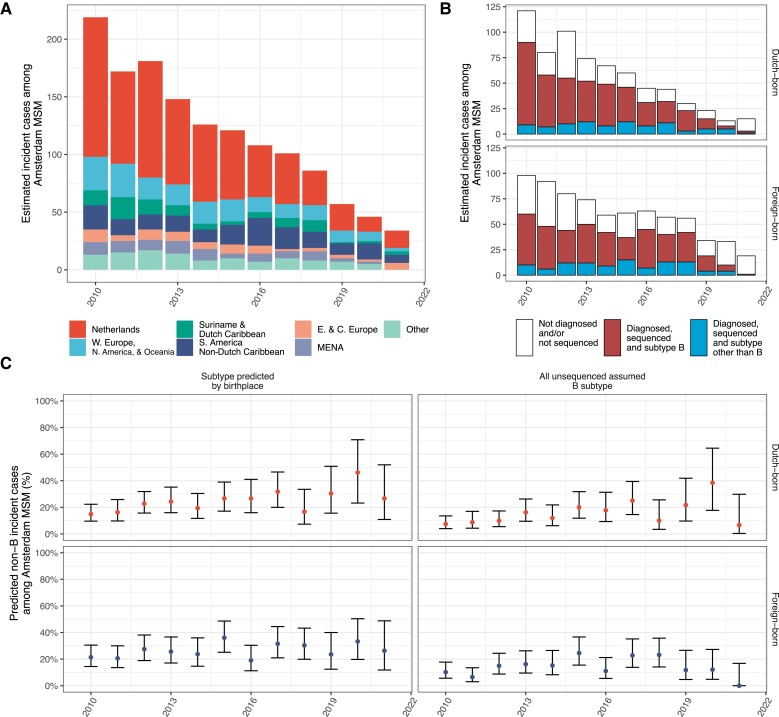
Time trends in human immunodeficiency virus (HIV) subtype frequencies among Amsterdam men who have sex with men (MSM). *A*, Estimated number of HIV incident Amsterdam MSM by calendar year of their median infection time estimates, stratified by place of birth. *B*, Estimated number of HIV incident Amsterdam MSM by calendar year of their median infection time estimates, and stratified by HIV subtype where available. *C*, Estimated proportion of non-B subtypes among HIV incident Amsterdam MSM by calendar year of median infection time estimates, accounting for unsequenced and undiagnosed individuals under 2 possible scenarios. The left scenario assumes that the acquisition of subtypes among unsequenced cases reflects proportions in sequenced cases by place of birth. The right scenario assumes that all unsequenced cases acquired a subtype B virus. Throughout, central estimates (dots) are shown along 95% Agresti-Coull confidence intervals (error bars). Abbreviations: MSM, men who have sex with men; E. & C. Europe, Eastern and Central Europe; MENA, Middle East and North Africa; N. America, North America; W. Europe, Western Europe.

There were 1335 MSM ATHENA participants who were ever resident in Amsterdam with an estimated infection date in 2010–2021, of whom 49% were born in and 51% outside the Netherlands (Table 1 and Figure 1*A*). Foreign-born Amsterdam MSM had significantly longer times to diagnosis than Dutch-born Amsterdam MSM (posterior median estimate, 11.5 years [95% posterior credible interval {CrI}, 10.1–13.0] vs 8.7 years [95% CrI, 7.7–9.9]).

**Table 1.** Characteristics of Amsterdam Men Who Have Sex With Men With Human Immunodeficiency Virus Enrolled Into the AIDS Therapy Evaluation in The Netherlands (ATHENA) Study

Characteristic	Amsterdam MSM	Sequenced Amsterdam MSM	Amsterdam MSM With Estimated Infection Date in 2010–2021	Sequenced Amsterdam MSM With Estimated Infection Date in 2010–2021
Total No.	6139	3367	1335	900
Age at estimated infection date				
15–24 y	811 (13)	466 (14)	206 (15)	129 (14)
25–34 y	2108 (34)	1216 (36)	483 (36)	322 (36)
35–44 y	1531 (25)	918 (27)	337 (25)	228 (25)
45–54 y	661 (11)	413 (12)	222 (17)	161 (18)
≥55 y	212 (3)	131 (4)	87 (7)	60 (7)
Unknown	816 (13)	223 (7)	0 (0)	0 (0)
Region of birth				
Netherlands	3282 (53)	1929 (57)	652 (49)	462 (51)
Western Europe, North America, & Oceania	1019 (17)	469 (14)	187 (14)	109 (12)
South America & non-Dutch Caribbean	610 (10)	292 (9)	163 (12)	112 (12)
Suriname & Dutch Caribbean	369 (6)	247 (7)	87 (6)	67 (7)
Middle East & North Africa	206 (3)	110 (3)	79 (6)	48 (5)
Eastern & Central Europe	157 (3)	78 (2)	65 (5)	37 (4)
Other	496 (8)	242 (7)	109 (8)	65 (7)
Subtype				
B	…	3022 (90)	…	704 (78)
01AE	…	80 (2)	…	43 (5)
02AG	…	66 (2)	…	24 (3)
A1	…	45 (1)	…	29 (3)
C	…	38 (1)	…	13 (1)
F1	…	20 (<1)	…	16 (2)
G	…	10 (<1)	…	6 (1)
D	…	7 (<1)	…	4 (<1)
06cpx	…	3 (<1)	…	1 (<1)
Other	…	76 (2)	…	60 (7)

Data are presented as No. (%).

Abbreviation: MSM, men who have sex with men.

Of these, 900 (67%) had an HIV pol sequence available. 840 (93%) Amsterdam MSM had subtype/CRF B, 01AE, 02AG, A1, C, F1, G, D, and 06cpx (Table 1), and we excluded 60 participants with uncommon/unclassifiable subtypes/CRFs. Sequence sampling fractions were similar across MSM born in different world regions (Supplementary Figure 2).

HIV subtype is a simple indicator into the origin of incident infections [8]. Most diagnosed and sequenced Dutch-born Amsterdam MSM with an estimated infection date in 2010–2021 had a subtype B virus (371/462 [80.3%]) (Figure 1*B*). More unexpectedly, the large majority of foreign-born Amsterdam MSM also had a subtype B virus (333/438 [76.0%]) in 2010–2021, suggesting predominant transmission from Western European or North American MSM among whom subtype B is most prevalent.

To characterize time trends, we next estimated the proportion of non-B incident cases among Dutch-born and foreign-born Amsterdam MSM in each calendar year using sequence data from 2010–2021. Figure 1*C* shows that the estimated proportion of incident cases with a non-B virus increased over time, even when assuming all unsequenced Amsterdam MSM acquired subtype B.

### Phylogenetic Data Exclude the Majority of Potential Sources

**Figure 2.**
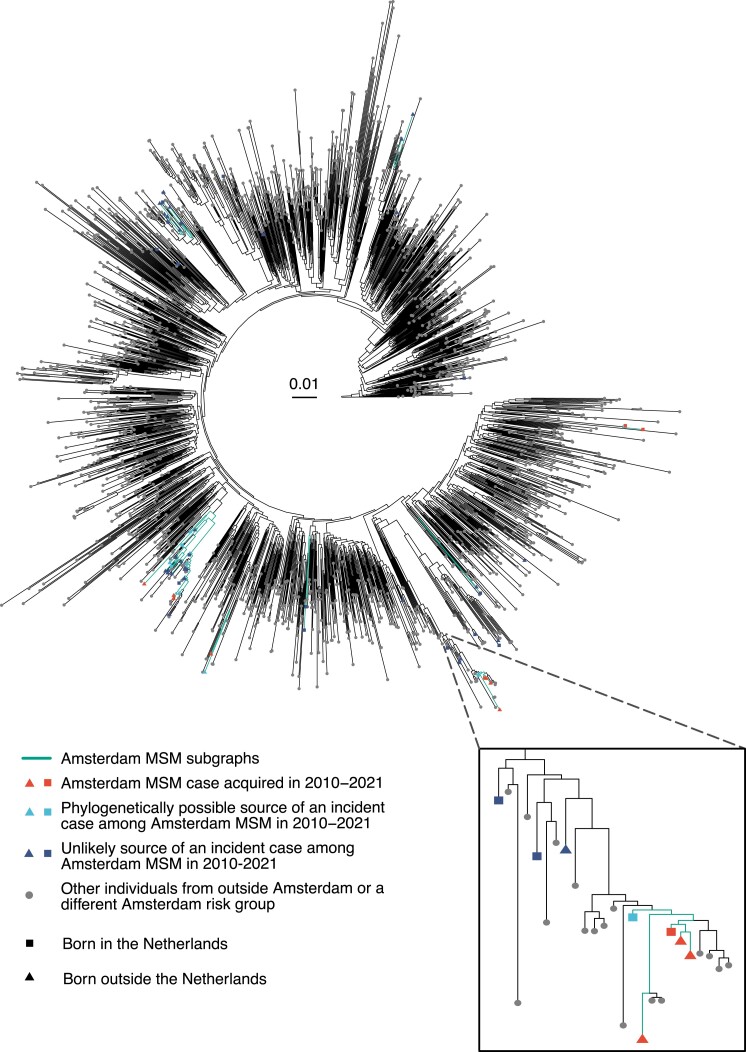
Phylogenetic tree for CRF 02AG sequences from Amsterdam men who have sex with men (MSM), ATHENA (AIDS Therapy Evaluation in the Netherlands) participants, and international background sequences. Amsterdam MSM subgraphs in the phylogenetic tree were reconstructed with phyloscanner and were identified as tips connected by continuous green branches with no change of color. Red tips denote incident cases among Amsterdam MSM estimated to have acquired their infection in 2010–2021. Light blue tips illustrate Amsterdam MSM who were in the same subgraph as the incident case, referred to as phylogenetically possible sources of each incident case; dark blue tips show Amsterdam MSM who are unlikely sources to these incident cases given phylogenetic, epidemiological, and clinical data; gray tips display non-Amsterdam or non-MSM cases. Squares represent Amsterdam MSM born in the Netherlands and triangles are those with a migration background.

To disentangle alternative hypotheses that could explain these trends in subtype frequencies, we conducted HIV phylogeographic analyses on the 840 sequenced Amsterdam MSM with estimated infection in 2010–2021, plus 2451 sequences from Amsterdam MSM with estimated infection before 2010, 1321 sequences from non-MSM risk groups in Amsterdam, 7119 sequences from ATHENA participants from the rest of the Netherlands, and 12 821 international sequences from the Los Alamos database that were closest to the ATHENA sequences from the major subtypes and CRFs among Amsterdam MSM (Figure 2 and Supplementary Table 1).

**Figure 3.**
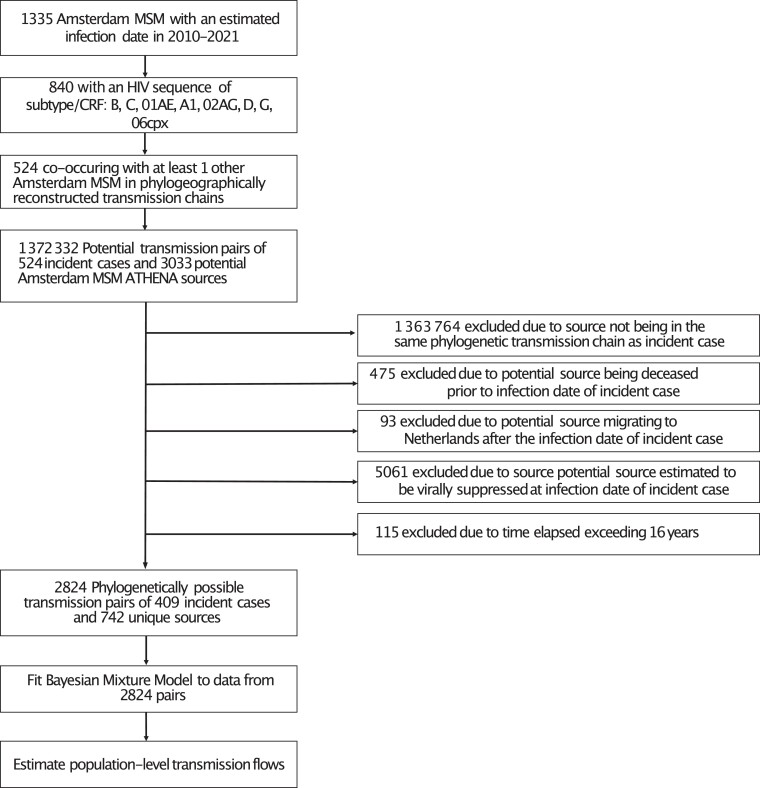
Source attribution analysis flowchart. Abbreviations: ATHENA, AIDS Therapy Evaluation in the Netherlands; CRF, circulating recombinant form; HIV, human immunodeficiency virus; MSM, men who have sex with men.

Across the 9 subtypes/CRFs, 376 (44.7%) Amsterdam MSM were phylogeographically rooted outside of Amsterdam MSM sequences and formed local phylogenetic transmission lineages of size [1], suggesting that these correspond to importations of new transmission lineages into Amsterdam or are part of locally ongoing transmission chains in which only 1 member was observed. In the absence of information on their source cases, we focused on the remaining 524 Amsterdam MSM with an estimated infection date in 2010–2021 who were part of phylogeographic Amsterdam MSM transmission chains. For these, we identified as potential sources 3033 Amsterdam MSM ATHENA participants who had a posterior median infection date before that of the incident case, forming 1 372 332 potential transmission pairs. We could exclude 99.8% pairs for whom the 2 individuals were not in the same phylogenetically inferred transmission chain, or who had other epidemiologic data rendering transmission highly improbable (Figure 3). Following exclusions, 115 incident cases had no plausible source remaining, leaving for source attribution analysis 2824 possible transmission pairs between 409 incident Amsterdam MSM and 742 unique possible sources in the same phylogeographic Amsterdam MSM transmission chain.

### Dutch-Born Amsterdam MSM Were Primary Sources of Locally Acquired Infections in 2010–2021

**Figure 4.**
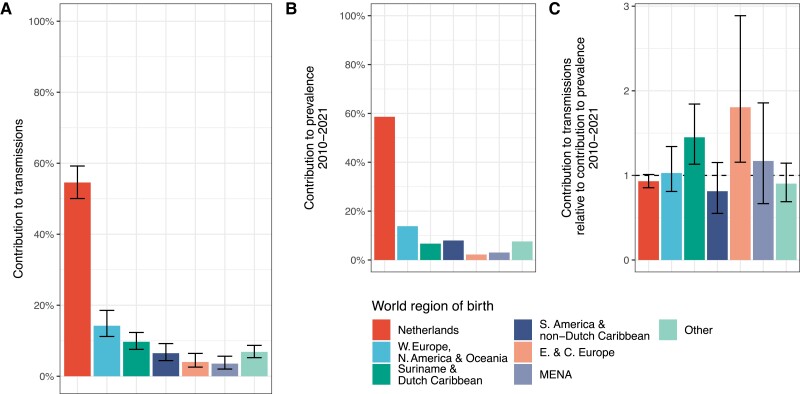
Sources of infections in Amsterdam men who have sex with men (MSM) who acquired HIV locally in Amsterdam transmission chains in 2010–2021. *A*, Estimated contributions of Dutch-born and foreign-born Amsterdam MSM to transmission to Amsterdam MSM in 2010–2021, with sources stratified by region of birth. Posterior median estimates (bars) are shown along with posterior 95% credible intervals (error bars). *B*, Estimated contributions of Dutch-born and foreign-born Amsterdam MSM to HIV prevalence among Amsterdam MSM in 2010–2021. *C*, Estimated contribution of Amsterdam MSM groups to transmission relative to their contribution to Amsterdam MSM with human immunodeficiency virus (HIV) in 2010–2021. Abbreviations: MSM, men who have sex with men; E. & C. Europe, Eastern and Central Europe; MENA, Middle East and North Africa; N. America, North America; W. Europe, Western Europe.

The clock-based source attribution model converged with no divergent transitions and fitted the data on 2824 possible transmission pairs well (Supplementary Figures 3 and 4). Overall, we found that an estimated 54.6% (95% CrI, 50.1%–59.2%) of local transmissions to Amsterdam MSM in 2010–2021 originated from Dutch-born MSM, which was very similar to their prevalence among Amsterdam MSM in the same time period (Figure 4). Most other groups of Amsterdam MSM born outside the Netherlands also contributed to transmission similar to their estimated prevalence among Amsterdam MSM, except for Amsterdam MSM born in Suriname and the Dutch Caribbean and Amsterdam MSM born in Eastern and Central Europe (Figure 4).

**Figure 5.**
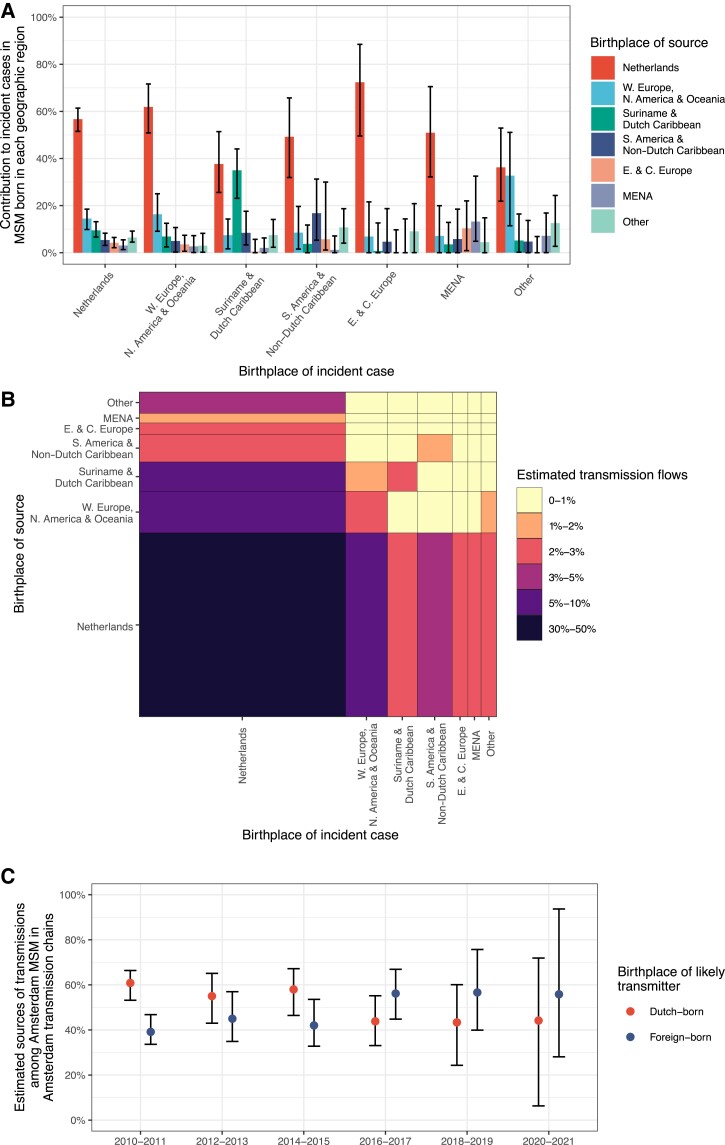
Sources of infections in Amsterdam men who have sex with men (MSM) who acquired HIV locally in Amsterdam transmission chains in 2010–2021, stratified by region of birth of both sources and incident cases and over time. *A*, Sources of infection in Amsterdam MSM who acquired infection through Amsterdam MSM transmission chains in 2010–2021. Region of birth of incident cases are shown on the x-axis, and the estimated contribution of transmission sources within each incident group is shown in color. Posterior median estimates (bars) are shown along with posterior 95% credible intervals (error bars). *B*, Estimated transmission flows between Amsterdam MSM subgroups in 2010–2021. The estimated posterior median proportion of transmission flows from the source group to the recipient group are visualized in colors. Cell widths correspond to the contribution of each Amsterdam MSM group to incidence, and cell heights correspond to the contribution of each Amsterdam MSM group to transmission sources. *C*, Sources of infection in Amsterdam MSM who acquired infection through Amsterdam MSM transmission chains in 2010–2021 by 2-year period. Year of acquired infection is shown on the x-axis in 2-year intervals, and the estimated contributions of transmission sources for incident cases among Dutch-born and foreign-born MSM are shown in color. Posterior median estimates (dots) are shown along with posterior 95% credible intervals (error bars). Abbreviations: MSM, men who have sex with men; E. & C. Europe, Eastern and Central Europe; MENA, Middle East and North Africa; N. America, North America; W. Europe, Western Europe.

We next characterized the sources of infections to each of the Amsterdam MSM subgroups stratified by region of birth. In 2010–2021, we estimated that Dutch-born Amsterdam MSM contributed the majority of transmissions to Amsterdam MSM born across all world regions of birth except for Amsterdam MSM born in Suriname and the Dutch Caribbean, for whom we estimated that similar proportions of transmission sources were Dutch-born MSM and MSM born in Suriname and the Dutch Caribbean (Figure 5*A*, Table 2). Further analyses reported in the Supplementary Material revealed a large phylogeographic transmission chain with MSM predominantly born in this region; however, this chain alone did not explain our findings, suggesting that the higher proportion of within-group transmission sources among Amsterdam MSM born in Suriname and the Dutch Caribbean appears to be more broadly sustained. Considering transmission flows between all Amsterdam MSM subgroups in 2010–2011, we found that the largest local transmission flows were between Dutch-born Amsterdam MSM (31.5% [95% CrI 28.4%–35.4%]), followed by Dutch-born MSM to MSM born in other countries in Western Europe, North America and Oceania, and vice versa (9.2% [95% CrI 6.9%–11.8%] and 8.1% [95% CrI 5.2%–10.3%], respectively) (Figure 5*B* and Table 2).

**Table 2.** Estimated Total Incident Cases Among Amsterdam Men Who Have Sex With Men in 2010–2021 and Sources of Locally Acquired Infections in Amsterdam Transmission Chains, Stratified by Place of Birth

Quantity	Birthplace of Incident Case						
	Netherlands	Western Europe, North America, & Oceania	Suriname & Dutch Caribbean	South America & Non-Dutch Caribbean	Eastern & Central Europe	Middle East & North Africa	Other
Estimated incident cases	673	192	178	91	81	70	114
No. of observed incident cases with sequence data	462	109	112	67	48	37	65
No. of phylogenetically and epidemiologically possible sources for incident cases born in each region	1391	392	283	293	130	108	227
Sources of Amsterdam MSM transmission for each recipient group, by birthplace of source^a^							
Netherlands	56.7% (51.5%–61.4%)	61.9% (50.9%–71.6%)	37.7% (25.6%–51.4%)	49.2% (31.9%–65.7%)	72.4% (49.6%–88.5%)	50.9% (32.2%–70.5%)	36.2% (21.9%–52.9%)
Western Europe, North America, & Oceania	14.5% (9.8%–18.5%)	16.3% (9.1%–25%)	7.4% (1.6%–14.3%)	8.5% (1.5%–19.6%)	6.9% (.0%–21.6%)	7.1% (.0%–20%)	32.7% (11.4%–51.1%)
Suriname & Dutch Caribbean	9.5% (6.6%–13.1%)	6.9% (2.4%–12.5%)	35.0% (23.1%–44.1%)	3.7% (.0%–11.8%)	0.7% (.0%–12.6%)	3.5% (.0%–12.9%)	5.2% (.2%–16.4%)
South America & non-Dutch Caribbean	5.4% (3.1%–8.3%)	5.0% (.2%–10.7%)	8.4% (3.3%–17.6%)	16.8% (5.3%–31.2%)	4.7% (.0%–18.7%)	5.8% (.0%–18.5%)	4.7% (.0%–13.7%)
Eastern & Central Europe	4.2% (2.1%–6.5%)	3.5% (.6%–7.4%)	0.5% (.0%–5.7%)	5.7% (1.1%–30%)	0.0% (.0%–9.7%)	10.3% (.9%–22%)	0.0% (.0%–6.9%)
Middle East & North Africa	3.0% (1.3%–5.5%)	2.7% (.1%–7.2%)	2.0% (.0%–6.2%)	1.2% (.0%–7.1%)	0.0% (.0%–14.4%)	13.2% (4.8%–32.5%)	7.2% (.0%–16.8%)
Other	6.4% (4.5%–9.2%)	3.0% (.2%–8.2%)	7.4% (2.3%–14.1%)	10.7% (4%–18.7%)	9.1% (.0%–20.8%)	4.4% (.0%–14.8%)	12.6% (2.7%–24.3%)
Amsterdam MSM transmission flows, by birthplace of source^b^							
Netherlands	31.5% (28.4%–35.4%)	9.2% (6.9%–11.8%)	2.7% (1.5%–3.9%)	3.7% (2.2%–5.2%)	3.0% (1.6%–4.5%)	2.1% (1.2%–4.0%)	2.1% (1.2%–3.1%)
Western Europe, North America, & Oceania	8.1% (5.2%–10.3%)	2.5% (1.3%–3.9%)	0.5% (.1%–1%)	0.6% (.1%–1.6%)	0.3% (.0%–1.0%)	0.3% (.0%–.9%)	2% (.5%–3.0%)
Suriname & Dutch Caribbean	5.3% (3.7%–7.3%)	1.0% (.4%–1.9%)	2.4% (1.5%–3.5%)	0.3% (.0%–.9%)	0.0% (.0%–.5%)	0.2% (.0%–.6%)	0.3% (.0%–1.0%)
South America & non-Dutch Caribbean	3.0% (1.7%–4.7%)	0.7% (.0%–1.6%)	0.6% (.2%–1.2%)	1.3% (.4%–2.3%)	0.2% (.0%–.8%)	0.2% (.0%–.9%)	0.3% (.0%–.8%)
Eastern & Central Europe	2.3% (1.2%–3.7%)	0.5% (.1%–1.1%)	0.5% (.2%–1.1%)	0.4% (.1%–2.5%)	0.0% (.0%–.4%)	0.4% (.0%–.9%)	0.0% (.0%–.4%)
Middle East & North Africa	1.7% (.7%–3.1%)	0.4% (.0%–1.1%)	0.1% (.0%–.4%)	0.1% (.0%–.6%)	0% (.0%–.5%)	0.6% (.2%–1.3%)	0.4% (.0%–1.0%)
Other	3.6% (2.5%–5.2%)	0.4% (.0%–1.2%)	0.5% (.2%–1.0%)	0.8% (.3%–1.5%)	0.4% (.0%–.9%)	0.2% (.0%–.6%)	0.8% (.1%–1.4%)

Data are presented as % (95% CrI).

Abbreviation: MSM, men who have sex with men.

^a^Columns sum to 100%.

^b^Rows and columns sum to 100%.

### Foreign-Born Amsterdam MSM Contribute Increasingly to Local Transmission

To further investigate the increasing trends in non-B subtypes among Amsterdam MSM (Figure 1), we characterized sources of infections for each 2-year period separately. Sample sizes of incident cases and possible sources were limited, declining from, respectively, 133 and 1465 in 2010–2011 to 11 and 37 in 2020–2021. Based on these data, we found increasing trends in local transmission from foreign-born Amsterdam MSM, and estimate that since 2016, foreign-born Amsterdam MSM contributed more to local HIV transmission in Amsterdam than Dutch-born MSM in the context of overall declining incidence (Figure 5*C*).

## DISCUSSION

Amsterdam has seen continued and sustained declines in HIV diagnoses and estimated new infections since 2010, coinciding with the introduction of now well-established test-and-treat strategies, and additional combination interventions introduced by H-TEAM that include preexposure prophylaxis (PrEP); innovative test-and-treat strategies at general practitioners, the Center for Sexual Health of the Public Health Service Amsterdam, and hospitals; and studies on motives and barriers for testing [29]. Of the remaining ongoing transmissions, the majority originate from within the city [4], and around a third are estimated to have been diagnosed within 6 months of infection [7], similar to MSM across Western Europe [30, 31]. Here, we integrated pathogen genomic data from Amsterdam, the rest of the Netherlands, and from international HIV sequence databases on all primary HIV subtypes and CRFs with demographic and clinical data to estimate the sources of infections in local, ongoing transmission chains among MSM who were ever resident in Amsterdam in 2010–2021. We considered in particular the transmission dynamics of foreign-born Amsterdam MSM, given the slower incidence declines in this group, and found that Dutch-born Amsterdam MSM were the predominant sources of transmission in all Amsterdam MSM populations who acquired their infection locally in 2010–2021, with the exception of Amsterdam MSM born in Suriname and the Dutch Caribbean, among whom we estimated that similar proportions of transmissions originated from MSM born in the Netherlands and those born in Suriname and the Dutch Caribbean. This finding is corroborated by foreign-born MSM predominantly presenting with a majority subtype B virus. Overall, these data from Amsterdam provide additional information into the transmission dynamics and infection sources to foreign-born MSM who acquire infection postmigration in Western Europe [2, 32, 33].

Considering previous investigations into the risk of outbreaks in the Netherlands driven by migrant populations [34], evidence from this study suggests that Amsterdam MSM with a migration background have overall not been the driver of local MSM transmissions since 2010. Yet, we found evidence for an increasing proportion of transmissions from foreign-born Amsterdam MSM. These shifts in transmission dynamics may be explained by differential uptake of HIV care and prevention services among Amsterdam MSM; for example, foreign-born MSM in the Netherlands are less likely to have heard of preexposure/postexposure prophylaxis (PEP/PrEP) and report experiencing more difficulties accessing healthcare than their native counterparts [35], and are more likely to present late, with qualitative data indicating one reason for this being fear of social stigma associated with a diagnosis [36].

Our findings should be considered in the context of the following limitations. First, ethnicity and age at migration were not recorded. It is possible that some of the Dutch-born transmission sources are of non-Dutch ethnicity [2, 37], and in this case local transmission dynamics among Amsterdam MSM may be more assortative than our analysis suggests. Second, we were unable to estimate the sources of locally acquired infections among Amsterdam residents reporting heterosexual contact due to small sample sizes and incomplete sequence availability. Considerably more assortative sexual mixing between foreign-born heterosexual individuals has been reported in the Netherlands [38–40] than among MSM, suggesting that transmission dynamics could be markedly different among heterosexuals. Third, while we attempted to account for potential biases due to sequence sampling heterogeneity, these adjustments are based on modeling assumptions and we cannot rule out that missing data may bias our findings. In particular, we cannot exclude the possibility that within-group transmission networks remained unsampled and that the proportion of within-group transmissions among foreign-born Amsterdam MSM is more similar to that seen among Amsterdam MSM born in Suriname and the Dutch Caribbean. Fourth, the process in characterizing possible transmission pairs is dependent on estimated infection dates and available demographic and clinical data, and thus may carry some uncertainty. We varied identification criteria in sensitivity analyses (Supplementary Material), suggesting our primary findings are robust to alterations of transmission pair selection criteria. Fifth, we characterized here the sources of city-level transmission, with a view on those transmissions that could be locally averted. The majority of Amsterdam MSM transmission chains originate from other areas in the Netherlands [4] where the contribution of Dutch-born individuals to the population is larger, suggesting that Dutch-born MSM could have a greater role in national-level transmission than our analysis indicates for Amsterdam MSM. Sixth, sample sizes were small in our temporal analysis, in particular for 2020–2021. Continued genomic surveillance at higher sequence sampling fractions will be essential to substantiate the growing proportions of local infections from foreign-born MSM that we identified from the available data.

The city of Amsterdam has already met 2025 UNAIDS 95-95-95 Fast-Track targets [7], and the focus of all stakeholders at the city level is now converging on reaching zero new HIV infections by 2026 [41]. In this effort, detailed phylogenetic analyses can provide helpful information to further target and refine prevention services, and to adapt these in the context of changes in transmission dynamics [4, 22, 42, 43]. In Amsterdam, foreign-born MSM continue to be associated with longer times from infection to diagnosis [4] and are more frequently presenting with a late-stage HIV infection at diagnosis [44]. However, recent programs across the Netherlands have sought to promote early diagnosis, for example, through home-based self-testing [45] and developing indicator-condition HIV testing [46, 47], with promising results. More widespread positive and inclusive prevention messaging to encourage knowing one's own HIV status and that of all sexual partners could further raise awareness and prevent local onward transmission among MSM. Our viral phylogenetic analyses indicate considerable potential for these and further strengthened prevention interventions among Amsterdam MSM because the majority of new MSM diagnoses continue to originate from local transmission chains among MSM and growing proportions of infections from foreign-born MSM.

## Supplementary Data

Supplementary materials are available at *The Journal of Infectious Diseases* online (http://jid.oxfordjournals.org/). Supplementary materials consist of data provided by the author that are published to benefit the reader. The posted materials are not copyedited. The contents of all supplementary data are the sole responsibility of the authors. Questions or messages regarding errors should be addressed to the author.

## Supplementary Material

jiae267_Supplementary_Data

## Notes

***Acknowledgments.*** We thank all contributors to ATHENA and Stichting HIV Monitoring (SHM), program staff and ATHENA participants; the Imperial College Research Computing Service (https://doi.org/10.14469/hpc/2232); and Zulip for sponsoring team communications through the Zulip Cloud Standard chat app.

***Author contributions.*** O. R. designed the study. O. R., P. R., and G. d. B. oversaw the study. A. v. S., D. B., and T. v. d. L. oversaw and performed data collection. A. B. and O. R. contributed to the analysis. A. B. and O. R. wrote the first draft. N. P., E. G. K., L. S., A. v. S., D. B., T. v. d. L., M. v. d. V., P. R., and G. d. B. discussed results and contributed to the revision of the final manuscript.

***Data availability.*** Pseudonymized individual-level patient and HIV consensus sequence data can be requested under a managed access policy from the HIV Monitoring Institutional Data Access Committee for researchers who meet the criteria for access to confidential data, which aligns with UNAIDS ethical guidelines. The process for accessing data is laid out in full at https://www.hiv-monitoring.nl/en/research-using-our-data/research-projects/submit-research-proposal. In brief, applicants can apply to receive data by submitting a concept sheet proposal in which they explain the research question and how they will mitigate potential risks to participant privacy to hiv.monitoring@amsterdamumc.nl. Each proposal must include a research summary, details of the project leader and participants, a research description, and the research design and plan. Following submission, the proposal will be reviewed by the HIV Monitoring Working Group. Final approval will be given by SHM's chair of the board. Prior to receiving data, the project leader must complete and return the Agreement for the use of SHM data (Inside EER or Outside EER).

***Code availability.*** All code to reproduce the analysis in this article is available from github.com/MLGlobalHealth/transmission.flows.by.birthplace.

***Financial support*.** This study received funding as part of the H-TEAM initiative from Aidsfonds (project number P29701); the Engineering and Physical Sciences Research Council (EP/X038440/1); and the Imperial College Department of Mathematics Cecilia Tanner Research Funding Scheme. The H-TEAM initiative is being supported by Aids fonds (grant numbers 2013169, P29701, and P60803); Stichting Amsterdam Dinner Foundation; Bristol-Myers Squibb (study number: AI424-541); Gilead Sciences Europe Ltd (grant number PA-HIV-PREP-16-0024); Gilead Sciences (protocol numbers CO-NL-276-4222, CO-US-276-1712, and CO-NL-985-6195); and the M·A·C AIDS Fund. The ATHENA database is maintained by SHM and supported by a grant from the Dutch Ministry of Health, Welfare and Sport through the Centre for Infectious Disease Control of the National Institute for Public Health and the Environment (RIVM).
