# Supplementary material for: Sources of Human Immunodeficiency Virus Infections Among Men Who Have Sex With Men With a Migration Background: A Viral Phylogenetic Case Study in Amsterdam, The Netherlands
Source: J Infect Dis. 2024 Jul 8;230(4):e881–94. doi: 10.1093/infdis/jiae267 (PMC11481325; doi:10.1093/infdis/jiae267)
Supplement: jiae267_Supplementary_Data [file jiae267_supplementary_data.pdf]

Supplementary Text to

Sources of HIV infections among MSM with a migration background: a viral phylogenetic case study in Amsterdam, the Netherlands

Blenkinsop et. al.

Contents

Supplementary Tables 2

Supplementary Figures 2

S1 Estimating HIV prevalence among Amsterdam MSM 6

S2 Distribution of subtypes among Amsterdam MSM 7

S3 Phylogenetic analyses 8

S4 Formulation of plausible transmission pairs 9

S5 Estimating transmission flows 10

S5.1 Bayesian Mixture Model 10

S5.2 Bayesian inference 12

S5.3 Target quantities 12

S5.4 Incorporating uncertainty in phylogenetic inference and time since infection 15

S6 Additional analyses of transmission dynamics among Amsterdam MSM born in Suriname & the Dutch Caribbean 15

S7 Sensitivity analyses 16

Supplementary Tables

Subtype	B	C	02AG	01AE	A1	G	D	F1	06cpx	Non-major subtypes and CRFs	Total (all)	Total considered in phylogenetic analysis
Amsterdam MSM	3,022 (90%)	38 (1%)	66 (2%)	80 (2%)	45 (1%)	10 (< 11%)	7 (< 11%)	20 (< 11%)	3 (< 11%)	76 (2%)	3,367	3,291
ATHENA (non-Amsterdam MSM)	5,940 (67%)	635 (7%)	780 (9%)	300 (3%)	338 (4%)	168 (2%)	106 (1%)	100 (1%)	73 (1%)	368 (4%)	8,808	8,440
International background (Total)	6050 (39%)	2254 (14%)	1082 (7%)	1050 (7%)	976 (6%)	482 (3%)	453 (3%)	329 (2%)	145 (1%)	2787 (18%)	15,608	12,821
Western Europe	2,279 (60%)	244 (6%)	302 (8%)	44 (1%)	136 (4%)	180 (5%)	25 (1%)	153 (4%)	44 (1%)	417 (11%)	3,824	3,407
Central Europe	782 (66%)	31 (3%)	25 (2%)	27 (2%)	73 (6%)	19 (2%)	18 (2%)	98 (8%)	3 (0%)	111 (9%)	1,187	1,076
Eastern Europe and Central Asia	60 (16%)	3 (1%)	2 (1%)	1 (< 1%)	6 (2%)	22 (6%)	0 (0%)	0 (0%)	3 (1%)	283 (74%)	380	97
South and South-East Asia	452 (22%)	161 (8%)	42 (2%)	930 (46%)	21 (1%)	21 (1%)	5 (< 1%)	2 (< 1%)	11 (1%)	372 (18%)	2,017	1,645
Africa	13 (< 1%)	1,599 (35%)	638 (14%)	5 (< 1%)	691 (15%)	221 (5%)	388 (8%)	11 (< 1%)	82 (2%)	986 (21%)	4,634	3,648
Middle East and North Africa	25 (14%)	8 (4%)	18 (10%)	9 (5%)	5 (3%)	1 (1%)	1 (1%)	1 (1%)	0 (0%)	117 (63%)	185	68
North America	1,936 (85%)	110 (5%)	48 (2%)	15 (1%)	42 (2%)	12 (1%)	8 (< 1%)	3 (< 1%)	1 (< 1%)	115 (5%)	2,290	2,175
Latin America and the Caribbean	420 (45%)	75 (8%)	6 (1%)	1 (< 1%)	0 (0%)	6 (1%)	3 (< 1%)	61 (7%)	0 (0%)	356 (38%)	928	572
Oceania	34 (85%)	0 (0%)	0 (0%)	5 (13%)	0 (0%)	0 (0%)	0 (0%)	0 (0%)	1 (3%)	0 (0%)	40	40
Unknown	49 (40%)	23 (19%)	1 (1%)	13 (11%)	2 (2%)	0 (0%)	5 (4%)	0 (0%)	0 (0%)	30 (24%)	123	93

Table S1: **Subtypes of Amsterdam MSM and other ATHENA sequences and subtypes of international background sequences, by geographic region of origin.**

Supplementary Figures

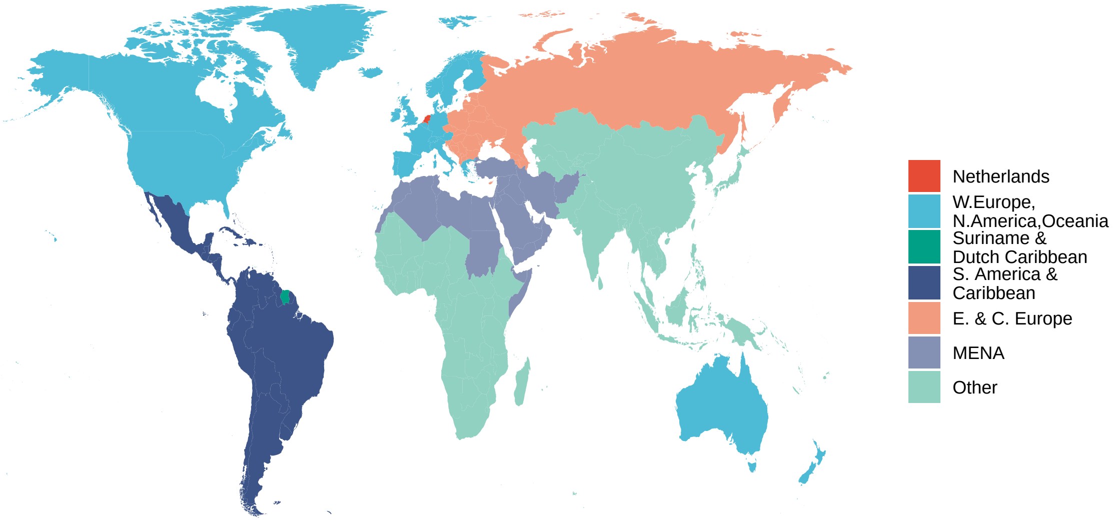

Figure S1: **Geographic regions for primary migrant groups among Amsterdam MSM.**

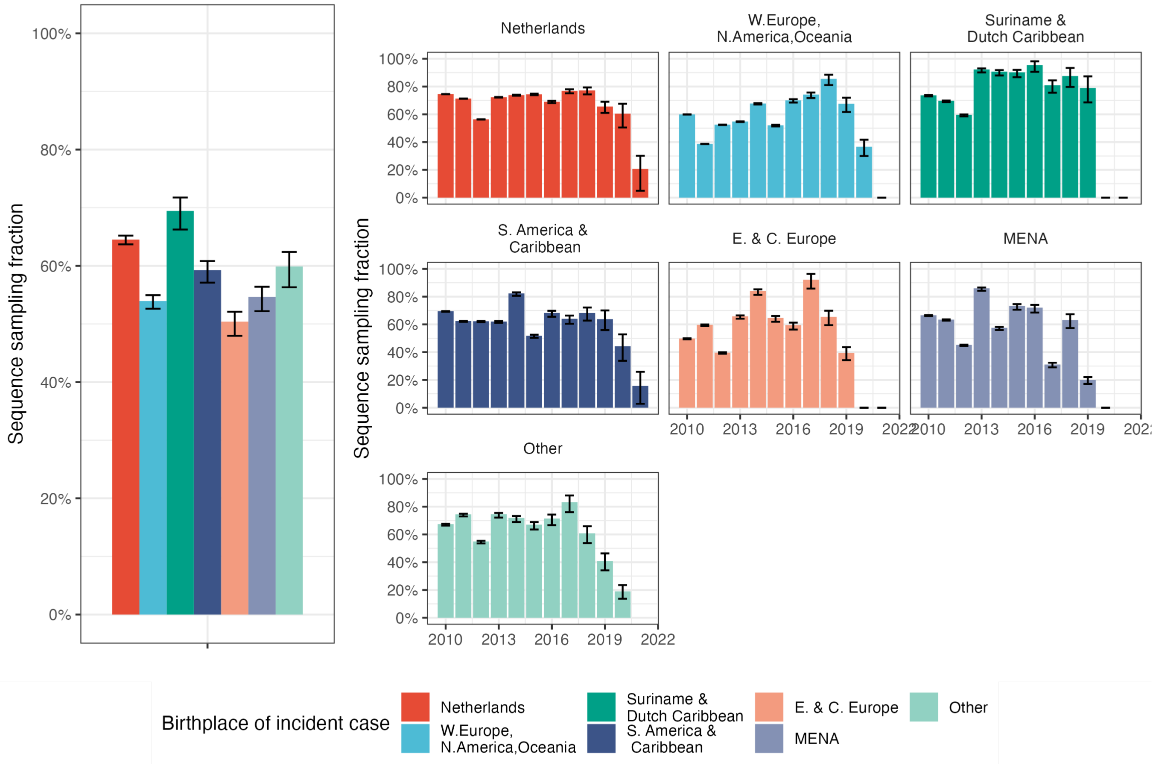

Figure S2: **Sequence sampling fractions by year, stratified by place of birth.** Proportion of estimated incident cases with a sequence available, by year of estimated HIV acquisition, with 95% credible intervals. The number of incident cases were estimated using bivariate linear mixed model (see text).

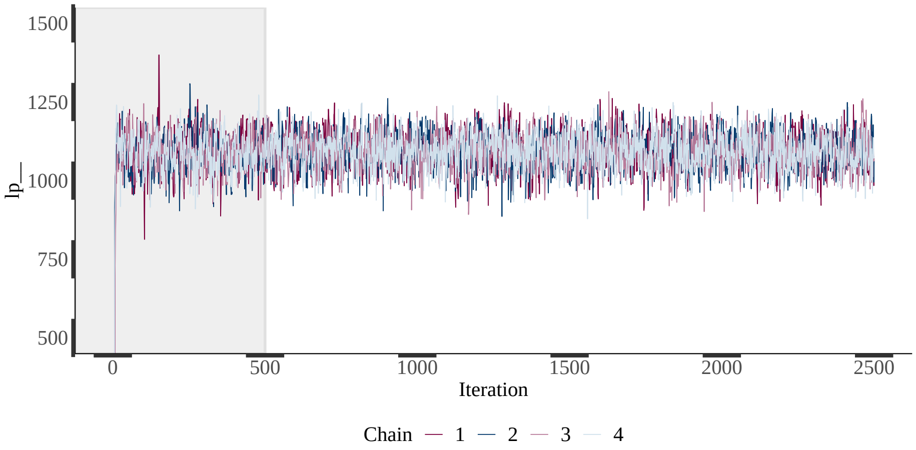

Figure S3: **Trace plot of parameter with the smallest effective sample size for the Bayesian Mixture Model used in the central analysis.**

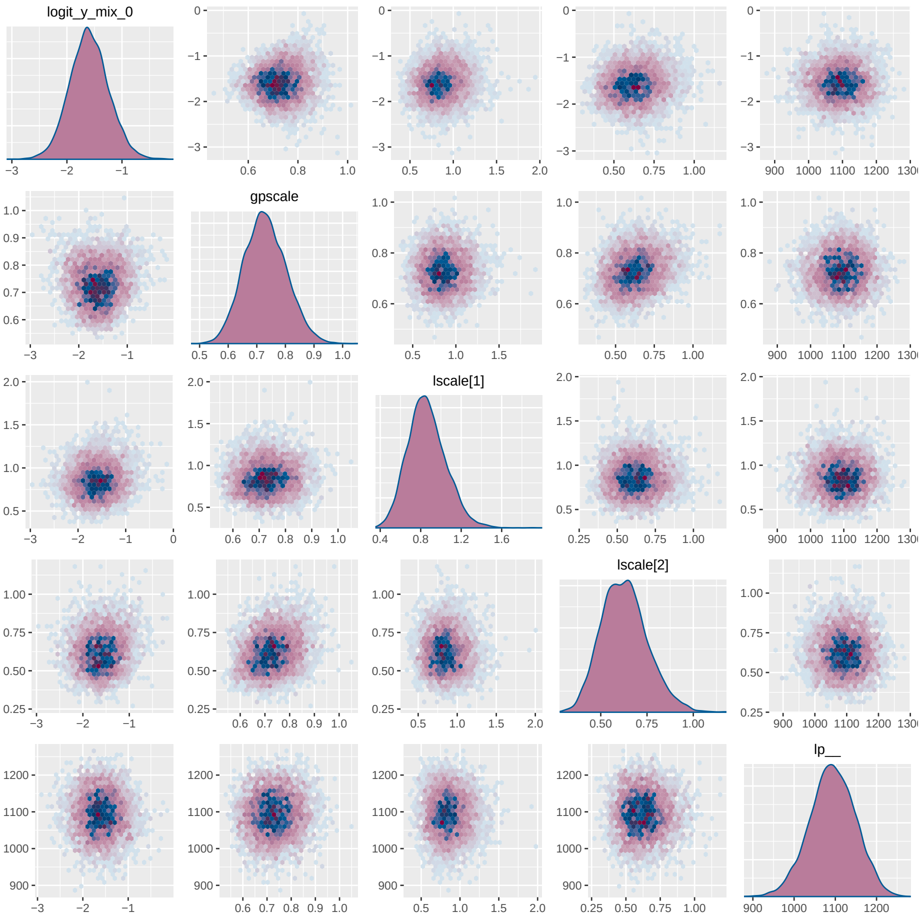

Figure S4: **Pairs plot of the estimated joint posterior density of all model parameters of the Bayesian Mixture Model used in the central analysis.** Coloured hexagons represent the binned 2D counts of posterior draws across monte carlo samples.

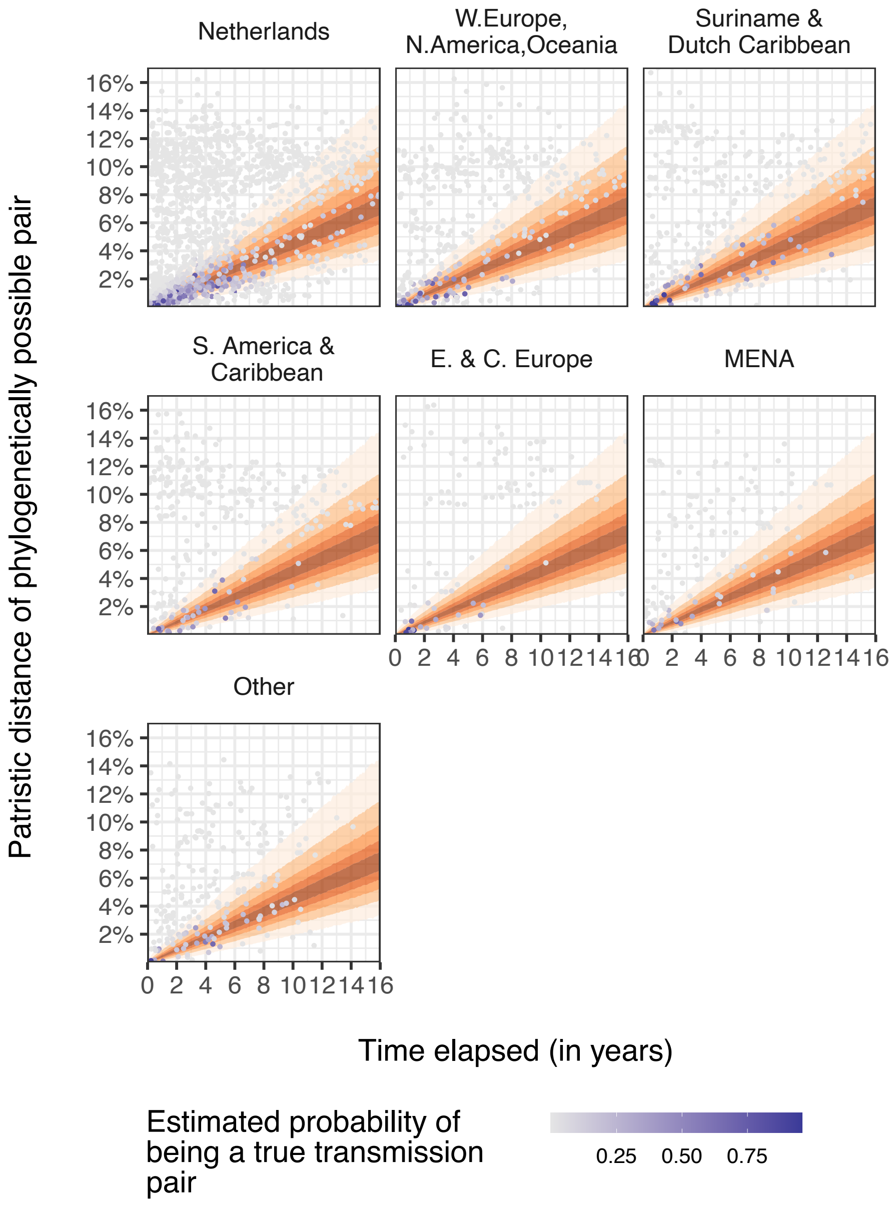

Figure S5: **Posterior median transmission pair probabilities for all phylogenetically possible pairs, by region of birth of the potential source in each pair.** Each point represents the patristic distance and estimated time elapsed of each phylogenetically possible transmission pair, and is coloured according to the posterior median transmission pair probability estimated by the model. Facets represent the geographic region of birth of the phylogenetically possible source in each pair. The orange bands represent the posterior quantiles of the evolutionary clock model, fitted to data from independent data on confirmed transmission pairs in Belgium (see text).

S1 Estimating HIV prevalence among Amsterdam MSM

To estimate population-level denominators for contextualising transmission flows, we considered all ATHENA participants since the start of data collection who ever resided in an Amsterdam postcode and self-reported as MSM, unless specified otherwise.

In order to obtain estimates of HIV prevalence among Amsterdam MSM stratified by place of birth, we followed methods previously described [1]. Specifically, we fitted a hierarchical Bayesian Weibull likelihood model to time since infection estimates by geographic place of birth for a sub-cohort of Amsterdam MSM estimated to have acquired HIV in 2010-2015, who are least likely subject to right censoring bias. Here, we stratified ATHENA study participants by geographic regions of birth, $\mathcal{K}$ = {Western Europe, North America & Oceania, Eastern and Central Europe, Suriname & the Dutch Caribbean, South America & the non-Dutch Caribbean, Middle East & North Africa (MENA), Other}; see also Supplementary Figure S1.

From the fitted model, we estimated the number of incident cases among Amsterdam MSM born in geographic region $k \in \mathcal{K}$ acquired in year $y \in \mathcal{Y} = \{1996,\dots,2021\}$, following previous methods [1]. Briefly, we estimate the posterior probability an individual who acquired HIV in year y was diagnosed by the database closure from the cumulative distribution function of the Weibull distribution, denoted by δ_{ky}. We divide the observed cases in each year and geographic region (N^{D}_{ky}) by δ_{ky} to obtain estimates of incidence, denoted N^{I}_{ky}, stratified by year and region of birth. We then estimated the number of people with HIV (PWH) in year y for MSM born in region k by summing over historical years since the start of ATHENA,

$$N^{\mathrm{PWH}}_{ky} = \sum_{i=1}^{y} N^{\mathrm{I}}_{ki} - N^{\mathrm{died}}_{ki}, \tag{S1}$$

where N^{died}_{ki} are the number of individuals reported to have died in year i.

We calculated the contribution of MSM born in each geographic region k towards total prevalence

over 2010-2021 as a weighted average as follows,

$$\pi_k^{\text{prevalence}} = \sum_{i=2010}^{2021} \frac{N_{ki}^{\text{PWH}}}{\sum_{k\in\mathcal{K}} N_{ki}^{\text{PWH}}}\, \omega_i, \tag{S2}$$

where $\omega_i = \frac{\sum_k N_{ki}^{\text{PWH}}}{\sum_k \sum_j N_{kj}^{\text{PWH}}}$ are the weights corresponding to the estimated total number of PWH among MSM in Amsterdam for year $i \in \mathcal{I} = \{2010, \ldots, 2021\}$.

S2 Distribution of subtypes among Amsterdam MSM

To assess whether the proportion of non-B subtypes has increased over time, we begin with the observed proportions from those MSM with a sequence (Supplementary Figure S6). To make population-level inferences, we estimated the subtypes of the unsequenced and/or undiagnosed MSM, based on the estimated total incident cases. Generally, the number of sequenced (N_{kis}^{Seq}) and unsequenced (N_{kis}^{U}) individuals sum to the total estimated incident cases,

$$N^{\text{I}} = \sum_{kis} (N_{kis}^{\text{Seq}} + N_{kis}^{\text{U}}), \tag{S3}$$

where $k \in \mathcal{K}$ denotes geographic region of birth, $i \in \mathcal{Y}$ denotes year of acquisition, and $s \in \mathcal{S} = \{\text{B, non-B}\}$ denotes HIV subtype. We used this to estimate the total number of unsequenced individuals among new cases in 1996-2021 by summing over the indices of N_{kis}^{U}.

In the most conservative scenario, we assume all unsequenced and/or undiagnosed MSM acquired a subtype B virus,

$$N_{kis}^{\text{U}} = \begin{cases} N_{ki}^{\text{I}} - \sum_{ki} N_{kis}^{\text{Seq}} & \text{when } s = \text{B} \\ 0 & \text{when } s = \text{Non-B}. \end{cases} \tag{S4}$$

We then asses whether we continue to observe an increase in the proportion of non-B viruses over time based on the total number of incident cases of subtype non-B (N_{kis}^{I}).

Alternatively, we may assume that the HIV subtypes of unsequenced and/or undiagnosed MSM follows similar trends to those observed in the sampled MSM. For this, we apply the proportion of MSM of subtype B, stratified by place of birth k and year i, denoted by $\pi_{ki\ \text{non-B}}$, to the total MSM

living with HIV minus those sequenced,

$$N^{\mathrm{U}}_{kis} = \pi_{ki\,\text{non-B}}(N^{\mathrm{I}}_{ki} - N^{\mathrm{Seq}}_{kis}). \tag{S5}$$

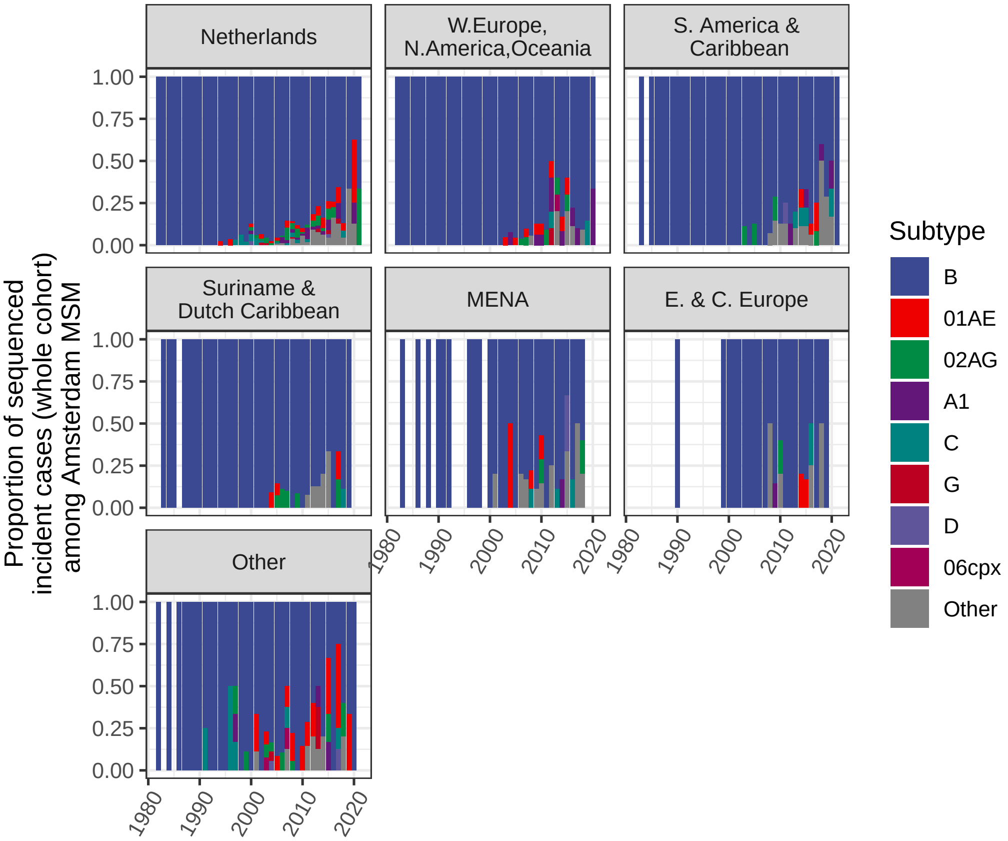

Figure S6: **Proportion of sequenced incident cases among Amsterdam MSM estimated to have been acquired from 1980 to 2021, by geographic region of birth and HIV subtype.**

S3 Phylogenetic analyses

Sequences were pairwise aligned to the reference sequence HXB2 using Virulign v1.0.1 [2], with MAFFT v7.453 [3] used to globally align any sequences which failed to align. Known drug-resistant mutations were masked using the R package big.phylo [4]. Sequences were subtyped by COMET v2.3 [5], with unknown or uncertain classifications verified with REGA v.3 [6]. 7,028 sequences from ATHENA participants from the Netherlands who were not Amsterdam-resident MSM were added as

phylogenetic background to the study, in addition to approximately 80,000 international background sequences from the Los Alamos sequence database. BLAST v2.10.0 [7] was used to select the 20 closest to each of the ATHENA sequences. The alignments for the Amsterdam and ATHENA sequences and for the closest background sequences were combined and trimmed, with a length of 1302 nucleotides. Sequence alignments were then split by each of the major subtypes or circulating recombinant forms (CRFs) circulating among Amsterdam MSM. Approximate maximum-likelihood phylogenetic trees were constructed for each subtype or CRF using single-precision FastTree v2.1.11 [8]. The Approximately Unbiased test [9] was used to compare topologies of trees inferred under the Jukes-Cantor nucleotide substitution model and the more flexible general-time-reversible (GTR) model for each subtype and CRF. The test found the latter model, with additional parameters, significantly improved fit for all subtypes and CRFs, and thus the GTR model was selected for phylogenetic inference. Ancestral state reconstruction was carried out on internal nodes of the estimated phylogenies using phyloscanner v1.8.0 [10], in which host states (defined as non-MSM Amsterdam risk groups or geographic regions) are attributed to lineages. The labels indicate where in the phylogeny the virus may have moved into Amsterdam MSM from one introduced case, and thus indicates geographic origins of local circulating lineages. Phylogenetic clusters were inferred as connected tips of Amsterdam MSM with the same assigned labels without a change of state along the branches.

S4 Formulation of plausible transmission pairs

To identify plausible transmission pairs, we begin by formulating all pairs in the same phylogenetic cluster. Next, we leverage additional data to exclude those which are not compatible with disease transmission via the following criteria,

1. Fit a loess curve to longitudinal viral load measurements for the source in each pair, and exclude sources who were likely virally suppressed and therefore uninfectious on the infection date of the recipient, based on a suppression threshold of <200 copies/ml [11, 12, 13].

2. Exclude pairs in which the probable source died prior to the putative infection date of the recipient.

3. For sources with a migration date, exclude pairs in which the probable source had not yet migrated to the Netherlands by the infection date of the recipient.

4. Exclude pairs with a time elapsed of over 16 years.

S5 Estimating transmission flows

S5.1 Bayesian Mixture Model

We follow methods described in detail in previous work [14], and here provide only a summary of the approach the Bayesian mixture model approach for phylodynamic source attribution. The performance of Bayesian mixture model approach to source attribution has previously been evaluated with simulation and was found to achieve acceptable estimation error under realistic simulation scenarios calibrated to real-world HIV prevention trial data, in comparison to approaches to source attribution based on patristic distance thresholds only [14].

The central idea of the mixture model is to classify observations (in this case potential transmission pairs) into two distinct densities, a signal component which characterises the data for a true transmission pair and a background component, which characterises the data for pairs which are unlinked epidemiologically. The density for the latter is straightforward, since unlinked pairs should exhibit no association between time elapsed and patristic distance. For the density corresponding to the true transmission pairs, it is necessary to know the expected genetic diversity between two individuals, given the time elapsed between them, corresponding to the evolutionary clock for HIV-1.

Data The setup, as outlined in main text, consists of phylogenetically and epidemiologically possible pairs of MSM, who are inferred to belong to the same local transmission network and have epidemiological and clinical data consistent with HIV transmission. Each pair is summarised by a two-dimensional vector comprising the time elapsed and the patristic distance between two individ-

uals in the inferred phylogeny. The former is calculated as the cumulative time elapsed between the estimated infection date of the incident case and the sequence sampling date of both the source and recipient. The latter quantity is calculated as the sum of the branch lengths connecting two individuals in the tree, corresponding to the number of nucleotide substitutions between them.

Estimating the evolutionary clock of HIV-1 We trained the signal component of the model on data from known transmission pairs to learn the relationship between time and genetic diversity. We leveraged published data from a study in Belgium in which a phylogenetic tree was validated with known transmission history for epidemiologically confirmed transmission pairs with a subtype C virus [15]. Following methods in [14], we constructed maximum-likelihood phylogenies using RAxML v.7.4.2 [16] and extracted pairwise patristic distances from each known transmission pair. We also calculated time elapsed corresponding to each patristic distance, using the date of the transmission event and sequence sampling dates. 2,807 data points were available from 7 transmission pairs, spanning genetic distances between $0.64 \times 10^{-2} - 6.9 \times 10^{-2}$ nucleotide substitutions per site and 0.70 - 16.5 years of time elapsed. We finally fitted a hierarchical model with a gamma likelihood to the time elapsed and patristic distances from the Belgian study, estimating random effects for each unique pair of individuals and hyper-parameters shared across pairs.

Two-component mixture model We developed a two-component Bayesian hierarchical mixture model, similar to those used widely for classification problems [17]. We embedded the gamma model within the signal component of the mixture model, fixing its hyper-parameters to the medians of their posterior predictive distribution from the evolutionary clock model fitted to the Belgian data and re-estimating the random effects for the new unseen pairs in the model from Amsterdam MSM. We assumed a 2D uniform distribution for the background component over time elapsed and patristic distance, with no parameters to be estimated. The model describes the likelihood that a particular combination of a patristic distance and time elapsed arose by chance through a background density or is compatible with a signal density, the evolutionary clock. In the mixture model, each observation

has a probability of belonging to the each of the two components, given by an unknown parameter to be estimated, in which the probabilities for the signal and background component must sum to one. We allowed the mixture probability to vary for each pair, and the probability of belonging to the signal component was modelled with a linear predictor, to estimate the probability a pair represents a true transmission pair given additional covariates from the incident case and putative source in each case. There is typically an association between ages of partners among MSM [18, 19], so the bivariate ages of the source and recipient in a pair is likely informative of being a true transmission pair. We therefore incorporated the age of both individuals within each pair on the estimated date of transmission to the incident case into the linear predictor for the pair-specific mixture parameters. Finally, we fitted the mixture model to the time elapsed, patristic distances and ages corresponding to each plausible pair of Amsterdam MSM.

S5.2 Bayesian inference

The model was fitted with `cmdstanr` v.2.28.1, with 4 chains of 2500 samples each, including a burn-in of 500. The model converged and mixed well, with a smallest effective sample size across the parameters of 3492, largest Rhat of 1.001 and no divergences.

S5.3 Target quantities

Probability that a phylogenetically observed pair represents a truly linked transmission pair. From the joint posterior, we estimated the probability that each phylogenetically possible pair with source i and recipient j belongs to the signal component of the mixture and thus represents a truly linked pair $\{c = 1\}$ (denoted $p_{ij}^{c=1}$). The probability is specified such that whilst each incident case, j, may have multiple phylogenetically possible sources, $i_1, \ldots, i_{L_j}$, no other point involving j can be simultaneously classified as a true transmission pair. The model also has the property that for any j the sum $\sum_i p_{ij}^{c=1}$ can be close to zero and never exceeds one, meaning the model can account for event the true source may not be among any of the observed, phylogenetically possible transmission

pairs involving j. The probability a pair i_u, j represent a true pair is therefore estimated by,

$$\begin{aligned}
&\rho_{i_u,j}|\boldsymbol{X} = \\
&\Big(\omega_{i_u,j}\, p(D_{i_uj}|T^e_{i_uj}, Z_{i_uj}=1) \prod_{v\neq u} (1-\omega_{i_v,j})\, p(D_{i_vj}|T^e_{i_vj}, Z_{i_vj}=0)\Big)\Big/ \\
&\Big[\sum_{w=1}^{n_j^{\mathcal{P}}} \Big(\omega_{i_w,j}\, p(D_{i_wj}|T^e_{i_wj}, Z_{i_wj}=1) \prod_{v\neq w} (1-\omega_{i_v,j})\, p(D_{i_vj}|T^e_{i_vj}, Z_{i_vj}=0)\Big) + \\
&\quad \prod_{v=1}^{n_j^{\mathcal{P}}} (1-\omega_{i_v,j})\, p(D_{i_vj}|T^e_{i_vj}, Z_{i_vj}=0)\Big].
\end{aligned} \tag{S6}$$

Transmission flows adjusted for incomplete sampling of incident cases. It is possible that not all incident cases since 2010 are sampled. To adjust for these, we first denote a partition of the study population with $\mathcal{A}$, and population groups in this partition by a, $b \in \mathcal{A}$. The number of incident cases born in geographic region a with an estimated date of HIV acquisition in year $y \in \mathcal{Y} = \{2010, \ldots, 2021\}$ is denoted by N^D_{ay}. Using time since infection estimates as previously described, we estimated the proportion of individuals who were undiagnosed by the end of follow-up at the start of 2022, and correspondingly denote the total number of incident cases, including those undiagnosed, by N^I_{ay}. The number of individuals who were diagnosed and have a sequence available is given by N^S_{ay}. We define the sequence sampling probability of each incidence case by geographic region of birth, a, by $\xi_{ay} = \frac{N^S_{ay}}{N^I_{ay}}$. We first estimated the population-level transmission counts originating from MSM born in region a to MSM born in region b,

$$Z_{ab} = \sum_{i\in a, j\in b} Z_{ij} = \sum_{i\in a}\sum_{j\in b} \frac{\rho_{ij}}{\xi_{bt(j)}} \tag{S7}$$

for all $a, b \in \mathcal{A}$, where $t(j)$ is the estimated year of HIV acquisition of recipient j. For subgroups in which $N^S_{bt} = 0$ but $N^I_{bt} > 0$, we calculate ξ_{bt} as $\frac{0.1}{N^I_{bt}}$, to allow estimates of Z_{ab} to be greater than zero in the event we did not sample any MSM. From this we calculate the population transmission flows originating from MSM born in region a through,

$$\delta_a = \Big(\sum_{b\in\mathcal{A}} Z_{ab}\Big)\Big/\Big(\sum_{c,d\in\mathcal{A}} Z_{cd}\Big), \tag{S8}$$

such that $\sum_a \delta_a = 1$. Just considering MSM born in a single world region in the denominator, the proportion of transmission flows among MSM born in region b originating from MSM born in region a is given by,

$$\delta_{ab} = Z_{ab} \Bigg/ \Bigg(\sum_{c \in \mathcal{A}} Z_{cb} \Bigg), \tag{S9}$$

such that $\sum_a \delta_{ab} = 1$. Finally, the population-level flows from MSM born in region a to MSM born in region b out of all transmissions is given by,

$$\pi_{ab} = Z_{ab} \Bigg/ \Bigg(\sum_{c,d \in \mathcal{A}} Z_{cd} \Bigg), \tag{S10}$$

such that $\sum_a \sum_b \pi_{ab} = 1$.

Transmission flows relative to prevalence. To contextualise the transmission flows by their relative population sizes, we calculate first the contribution of MSM born in each world region to prevalence as a weighted sum over the years 2010-2021. The weights for the years are calculated as,

$$\omega_y = \frac{\sum_{a \in \mathcal{A}} N_{ay}^{\mathrm{PWH}}}{\sum_{y=2010}^{2021} \sum_{a \in \mathcal{A}} N_{ay}^{\mathrm{PWH}}}. \tag{S11}$$

Then, the contributions of each world region is,

$$p_a = \sum_{y=2010}^{2021} \omega_y \frac{N_{ay}^{\mathrm{PWH}}}{\sum_{a \in \mathcal{A}} N_{ay}^{\mathrm{PWH}}}. \tag{S12}$$

Then the relative flows are calculated as,

$$\phi_a = \frac{\delta_a}{p_a}, \tag{S13}$$

for each Monte Carlo sample.

S5.4 Incorporating uncertainty in phylogenetic inference and time since infection

To account for uncertainty in phylogenetic inference by bootstrap sampling subtype-specific sequence alignments 50 times and repeating our inferences derived from these bootstrap sequence alignments. We further accounted for uncertainty in time since infection estimates by sampling the time since infection for participants in each of the 50 bootstrap replicate trees from a uniform distribution over the posterior 95% credible interval from the Bayesian model for estimating time since infection. We reformulated phylogenetically likely transmission pairs and fitted the model independently to the 50 sets of pairs, aggregating and summarising their posterior transmission flows by their medians and 95% quantiles.

S6 Additional analyses of transmission dynamics among Amsterdam MSM born in Suriname & the Dutch Caribbean

We estimated in the central analysis that among Amsterdam MSM born in Suriname & the Dutch Caribbean as many transmissions originated from other MSM born in the same region as from Dutch-born MSM.

We investigated this further and identified there was one large phylogenetic subgraph with 11 MSM born in Suriname & the Dutch Caribbean, of who five were incident cases acquired since 2010. Excluding this subgraph, we re-estimated the transmission flows towards MSM born in Suriname & the Dutch Caribbean and found that 28% of transmissions were still attributed to have their source in other MSM born in Suriname & the Dutch Caribbean. This corresponded to a reduction of 8% in estimated transmission flows, and suggest that the identified large phylogenetic subgraph alone does not explain the estimated, increased within-group transmission flows among Amsterdam MSM born in Suriname & the Dutch Caribbean.

We then considered all phylogenetically possible within-group transmission pairs, and summed the density values of the mixture model component that captures the likelihood of each observed

pair being a true transmission pair as an empirical measure of evidence of within-group transmission flows (Supplementary Figure S7). The analysis indicated that MSM born in Suriname & the Dutch Caribbean had the second most frequent counts after Dutch-born pairs, suggesting there were many phylogenetically and epidemiologically plausible pairs between MSM born in this region that are compatible with direct transmission between them, and confirming that the reconstructed phylogenetic subgraph alone did not explain the estimated, increased within-group transmission flows among Amsterdam MSM born in Suriname & the Dutch Caribbean.

S7 Sensitivity analyses

We performed a series of sensitivity analyses to investigate the impact of each of the exclusion criteria shown in Figure 3 on the transmission flow estimates. Specifically, we re-fitted the model omitting each exclusion criteria in turn and estimated sources of infection for the revised set of potential transmission pairs. Table S2 compares the estimated transmission flows among Amsterdam MSM originating from each of the seven geographic regions when the model was fitted to pairs applying all exclusion criteria (the central analysis), and by omitting each exclusion criteria using epidemiological and clinical data in turn. Across the sensitivity analyses, we observed similar proportions of transmissions that originated from Amsterdam MSM born in the different geographic regions considered, suggesting in turn that our findings are robust with regards to the additional exclusion criteria based on epidemiologic data that we used in this analysis.

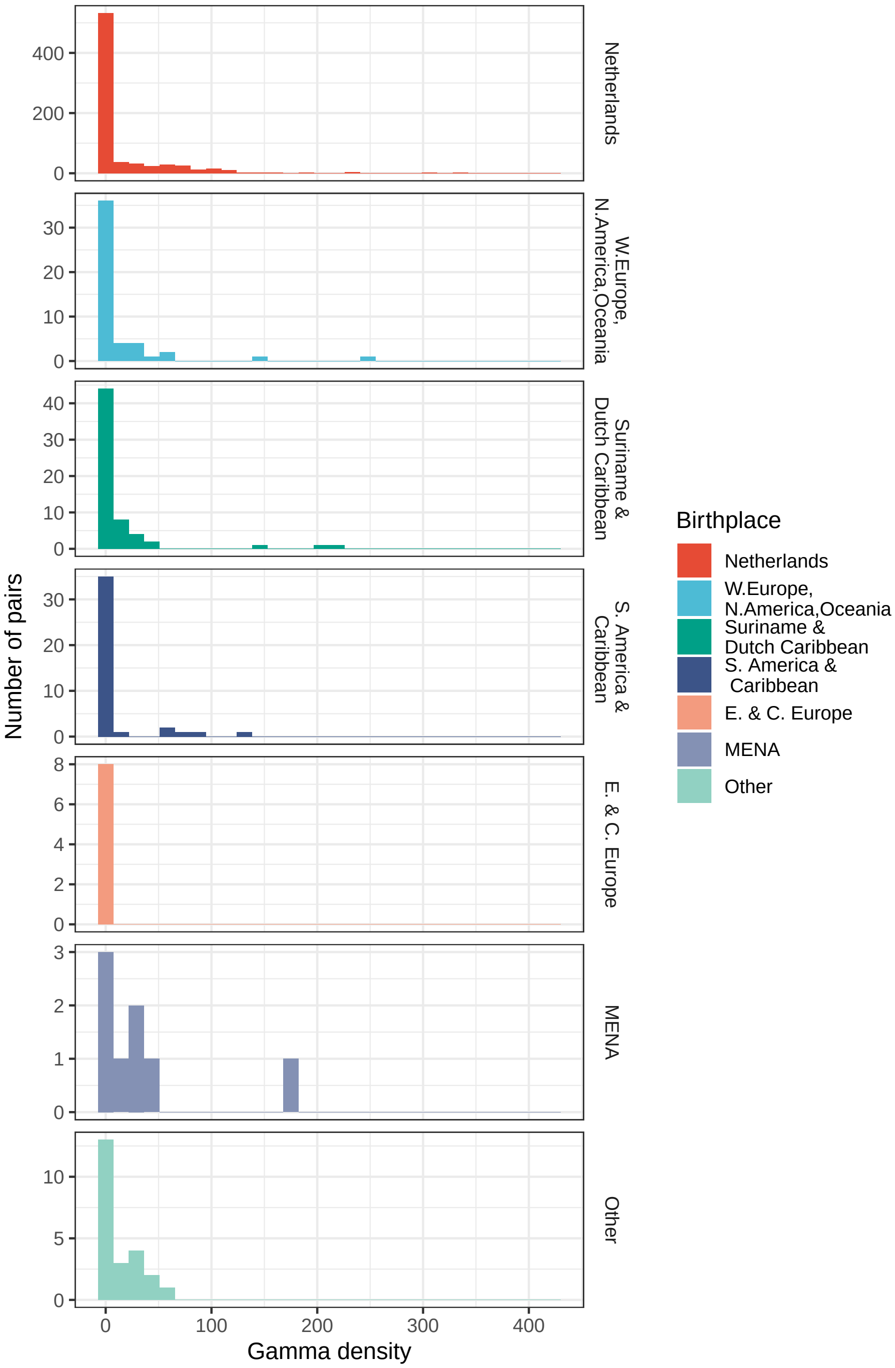

Figure S7: **Histogram of the density values of the mixture model component that captures the likelihood of each observed pair being a true transmission pair gamma densities, for phylogenetically possible transmission pairs with both individuals born in the same geographic region.**

		Estimated transmission flows originating from MSM born in each region						
Exclusion criteria omitted	Number of pairs	Netherlands	W.Europe, N.America, Oceania	Suriname & Dutch Caribbean	S. America & non-Dutch Caribbean	E. & C. Europe	Middle East & N. Africa	Other
None (central analysis)	2,824	56%	15%	10%	7%	4%	3%	6%
Date of death of source incompatible with transmission	3,203	57%	14%	10%	6%	4%	3%	6%
Migration date of source dates incompatible with transmission	2,917	54%	15%	11%	7%	4%	3%	6%
Viral load of source incompatible with transmission	7,232	58%	12%	10%	6%	3%	4%	7%
Time elapsed exceeding 16 years	2,939	56%	14%	10%	7%	4%	3%	6%

Table S2: **Sensitivity analyses on the impact of the additional epidemologic exclusion criteria on transmission flow estimates.**

References

[1] Blenkinsop, A. *et al.* Estimating the potential to prevent locally acquired HIV infections in a UNAIDS Fast-Track City, Amsterdam. *eLife* **11**, e76487 (2022). URL https://doi.org/10.7554/eLife.76487.

[2] Libin, P. J. K., Deforche, K., Abecasis, A. B. & Theys, K. VIRULIGN: fast codon-correct alignment and annotation of viral genomes. *Bioinformatics* **35**, 1763–1765 (2018). URL https://doi.org/10.1093/bioinformatics/bty851. https://academic.oup.com/bioinformatics/article-pdf/35/10/1763/28604627/bty851.pdf.

[3] Katoh, K. & Standley, D. M. MAFFT Multiple Sequence Alignment Software Version 7: Improvements in Performance and Usability. *Mol Biol Evol* **30**, 772–780 (2013). URL https://doi.org/10.1093/molbev/mst010. https://academic.oup.com/mbe/article-pdf/30/4/772/6420419/mst010.pdf.

[4] Ratmann, O. big.phylo (2020). URL https://github.com/olli0601/big.phylo.

[5] Struck, D., Lawyer, G., Ternes, A.-M., Schmit, J.-C. & Bercoff, D. P. COMET: adaptive context-based modeling for ultrafast HIV-1 subtype identification. *Nucleic Acids Res* **42**, e144–e144 (2014). URL https://doi.org/10.1093/nar/gku739. https://academic.oup.com/nar/article-pdf/42/18/e144/17423386/gku739.pdf.

[6] Pineda-Peña, A.-C. *et al.* Automated subtyping of HIV-1 genetic sequences for clinical and surveillance purposes: Performance evaluation of the new rega version 3 and seven other tools. *Infect Genet Evol* **19**, 337–348 (2013). URL https://www.sciencedirect.com/science/article/pii/S1567134813001810.

[7] Altschul, S. F., Gish, W., Miller, W., Myers, E. W. & Lipman, D. J. Basic local alignment search tool **215**, 403–410 (1990). URL https://doi.org/10.1016/s0022-2836(05)80360-2.

[8] Price, M. N., Dehal, P. S. & Arkin, A. P. Fasttree 2 – approximately maximum-likelihood trees for large alignments. *PLOS ONE* **5**, 1–10 (2010). URL https://doi.org/10.1371/journal.pone.0009490.

[9] Shimodaira, H. An approximately unbiased test of phylogenetic tree selection. *Syst Biol* **51**, 492–508 (2002). URL http://dx.doi.org/10.1080/10635150290069913.

[10] Wymant, C. *et al.* PHYLOSCANNER: Inferring transmission from within- and between-host pathogen genetic diversity. *Mol Biol Evol* **35**, 719–733 (2018). URL http://creativecommons.

[11] Rodger, A. J. *et al.* Sexual activity without condoms and risk of HIV transmission in serodifferent couples when the HIV-positive partner is using suppressive antiretroviral therapy. *JAMA* **316**, 171 (2016). URL https://doi.org/10.1001/jama.2016.5148.

[12] Bavinton, B. *et al.* Hiv treatment prevents hiv transmission in maleserodiscordant couples in australia, thailand and brazil. In *9th IAS Conference on HIV Science, Paris, France*, vol. TUAC0506LB (2017).

[13] Rodger, A. J. *et al.* Risk of hiv transmission through condomless sex in serodifferent gay couples with the hiv-positive partner taking suppressive antiretroviral therapy (partner): final results of a multicentre, prospective, observational study. *Lancet* **393**, 2428–2438 (2019). URL http://dx.doi.org/10.1016/S0140-6736(19)30418-0.

[14] Blenkinsop, A. *et al.* Bayesian mixture models for phylogenetic source attribution from consensus sequences and time since infection estimates — arxiv.org ((pre-print)). URL https://doi.org/10.48550/arXiv.2304.06353. [Accessed 12-Jun-2023].

[15] Vrancken, B. *et al.* The genealogical population dynamics of HIV-1 in a large transmission chain: bridging within and among host evolutionary rates. *PLoS Comput Biol* **10**, e1003505 (2014).

[16] Stamatakis, A. Raxml version 8: a tool for phylogenetic analysis and post-analysis of large phylogenies. *Bioinformatics* **30**, 1312–1313 (2014).

[17] McLachlan, G. J., Lee, S. X. & Rathnayake, S. I. Finite mixture models. *Annu Rev Stat Appl* **6**, 355–378 (2019). URL `https://doi.org/10.1146%2Fannurev-statistics-031017-100325`.

[18] Kusejko, K. *et al.* Inferring the age difference in HIV transmission pairs by applying phylogenetic methods on the HIV transmission network of the swiss HIV cohort study. *Virus Evol* **4** (2018). URL `https://doi.org/10.1093%2Fve%2Fvey024`.

[19] Dennis, A. M. *et al.* HIV-1 transmission clustering and phylodynamics highlight the important role of young men who have sex with men. *AIDS Res Hum Retroviruses* **34**, 879–888 (2018). URL `https://doi.org/10.1089/aid.2018.0039`.
